# Supplementary material for: In Vitro Evaluation of the Antimicrobial Activity of Eighteen Essential Oils Against Gram-Positive and Gram-Negative Bacteria in Two Different Growth Media
Source: Pathogens. 2025 Nov 29;14(12):1216. doi: 10.3390/pathogens14121216 (PMC12736375; doi:10.3390/pathogens14121216)
Supplement: Supplementary file 1 [file pathogens-14-01216-s001.zip › pathogens-3961034-supplementary.pdf]

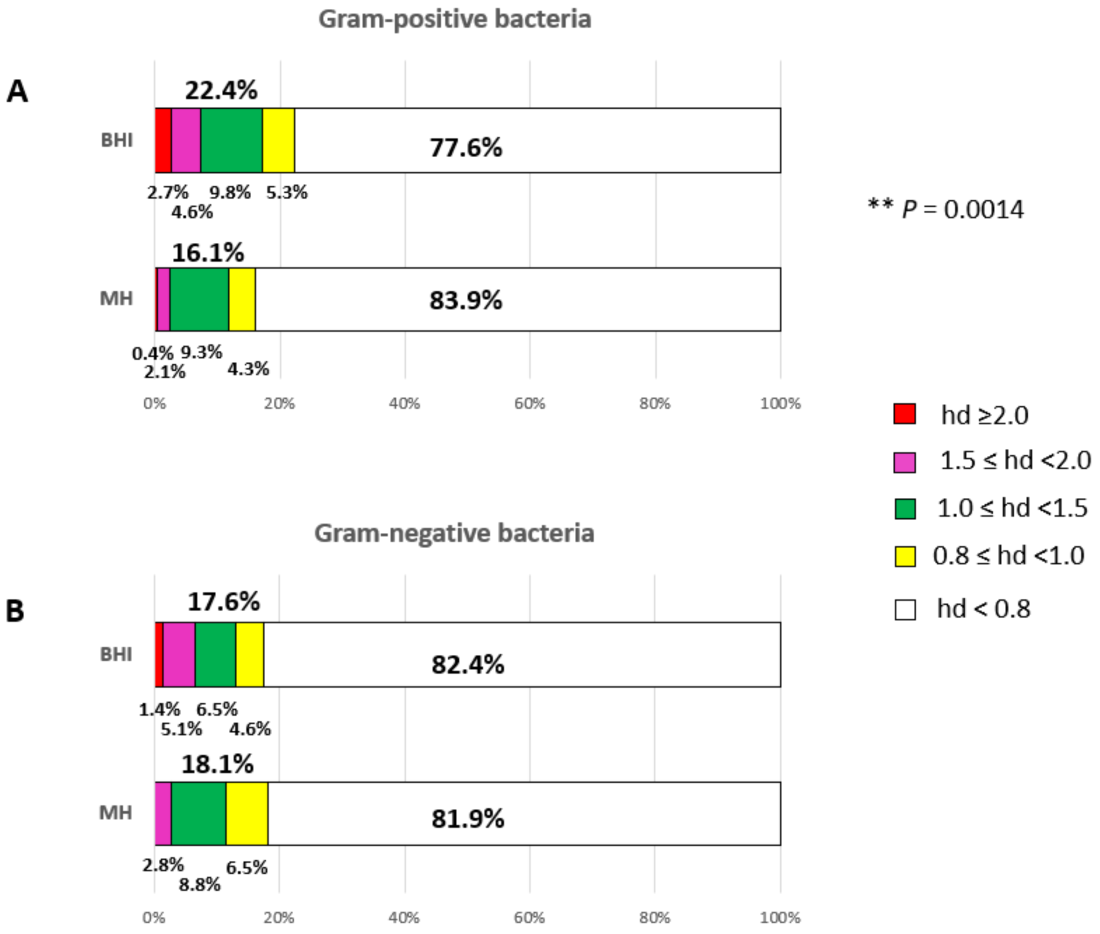

Figure S1. The overall efficacy of EOs by assessing the frequency of efficacious bacterial growth inhibition on BHI and MH agar plates after the three time points considered: 24 hours, 48 hours and 7 days. Panel A, antimicrobial power of EOs against Gram-positive bacteria. Panel B, antimicrobial power of EOs against Gram-negative bacteria. Sub-bars indicating the frequency of very high (red), high (magenta), moderate (green), low (yellow) and very low/null (white) antimicrobial activities are shown. The efficacy of EOs is measured by the diameter of the inhibition zones (haloes) in centimetres after incubation.

Printout S1. The main effect of EO exposure on mean hd measurements in Gram-positive bacteria. Between-group analysis for the effect of EO exposure was performed by one-way ANOVA, followed by a post-hoc Tukey's multiple comparisons test.

ANOVA results | Multiple comparisons

Ordinary one-way ANOVA<br>Multiple comparisons						
Number of families	1					
Number of comparisons per family	153					
Alpha	0.05					
**Tukey's multiple comparisons test**	**Mean Diff.**	**95.00% CI of diff.**	**Below threshold?**	**Summary**	**Adjusted P Value**	
cinnamon vs. bergamot	0.5300	0.2451 to 0.8149	Yes	****	<0.0001	A-B
cinnamon vs. lemon	0.4420	0.1571 to 0.7269	Yes	****	<0.0001	A-C
cinnamon vs. cumin	0.3880	0.1031 to 0.6729	Yes	***	0.0003	A-D
cinnamon vs. juniper	0.1170	-0.1679 to 0.4019	No	ns	0.9940	A-E
cinnamon vs. lavender	0.2880	0.003080 to 0.5729	Yes	*	0.0443	A-F
cinnamon vs. laurel	0.3890	0.1041 to 0.6739	Yes	***	0.0003	A-G
cinnamon vs. tea tree	-0.2980	-0.5829 to -0.01308	Yes	*	0.0295	A-H
cinnamon vs. peppermint	0.08200	-0.2029 to 0.3669	No	ns	>0.9999	A-I
cinnamon vs. myrtle	0.4710	0.1861 to 0.7559	Yes	****	<0.0001	A-J
cinnamon vs. basil	0.4890	0.2041 to 0.7739	Yes	****	<0.0001	A-K
cinnamon vs. oregano	-1.107	-1.392 to -0.8221	Yes	****	<0.0001	A-L
cinnamon vs. black pepper	0.3770	0.09208 to 0.6619	Yes	***	0.0006	A-M
cinnamon vs. rosemary	0.4440	0.1591 to 0.7289	Yes	****	<0.0001	A-N
cinnamon vs. sage	0.4800	0.1951 to 0.7649	Yes	****	<0.0001	A-O
cinnamon vs. clove	-0.1880	-0.4729 to 0.09692	No	ns	0.6746	A-P
cinnamon vs. thyme	-0.7570	-1.042 to -0.4721	Yes	****	<0.0001	A-Q
cinnamon vs. ginger	0.5300	0.2451 to 0.8149	Yes	****	<0.0001	A-R
bergamot vs. lemon	-0.08800	-0.3729 to 0.1969	No	ns	0.9998	B-C
bergamot vs. cumin	-0.1420	-0.4269 to 0.1429	No	ns	0.9568	B-D
bergamot vs. juniper	-0.4130	-0.6979 to -0.1281	Yes	****	<0.0001	B-E
bergamot vs. lavender	-0.2420	-0.5269 to 0.04292	No	ns	0.2153	B-F
bergamot vs. laurel	-0.1410	-0.4259 to 0.1439	No	ns	0.9595	B-G
bergamot vs. tea tree	-0.8280	-1.113 to -0.5431	Yes	****	<0.0001	B-H
bergamot vs. peppermint	-0.4480	-0.7329 to -0.1631	Yes	****	<0.0001	B-I
bergamot vs. myrtle	-0.05900	-0.3439 to 0.2259	No	ns	>0.9999	B-J
bergamot vs. basil	-0.04100	-0.3259 to 0.2439	No	ns	>0.9999	B-K
bergamot vs. oregano	-1.637	-1.922 to -1.352	Yes	****	<0.0001	B-L
bergamot vs. black pepper	-0.1530	-0.4379 to 0.1319	No	ns	0.9179	B-M
bergamot vs. rosemary	-0.08600	-0.3709 to 0.1989	No	ns	0.9999	B-N
bergamot vs. sage	-0.05000	-0.3349 to 0.2349	No	ns	>0.9999	B-O
bergamot vs. clove	-0.7180	-1.003 to -0.4331	Yes	****	<0.0001	B-P
bergamot vs. thyme	-1.287	-1.572 to -1.002	Yes	****	<0.0001	B-Q
bergamot vs. ginger	0.000	-0.2849 to 0.2849	No	ns	>0.9999	B-R
lemon vs. cumin	-0.05400	-0.3389 to 0.2309	No	ns	>0.9999	C-D
lemon vs. juniper	-0.3250	-0.6099 to -0.04008	Yes	**	0.0089	C-E
lemon vs. lavender	-0.1540	-0.4389 to 0.1309	No	ns	0.9135	C-F
lemon vs. laurel	-0.05300	-0.3379 to 0.2319	No	ns	>0.9999	C-G
lemon vs. tea tree	-0.7400	-1.025 to -0.4551	Yes	****	<0.0001	C-H
lemon vs. peppermint	-0.3600	-0.6449 to -0.07508	Yes	**	0.0015	C-I
lemon vs. myrtle	0.02900	-0.2559 to 0.3139	No	ns	>0.9999	C-J
lemon vs. basil	0.04700	-0.2379 to 0.3319	No	ns	>0.9999	C-K
lemon vs. oregano	-1.549	-1.834 to -1.264	Yes	****	<0.0001	C-L
lemon vs. black pepper	-0.06500	-0.3499 to 0.2199	No	ns	>0.9999	C-M
lemon vs. rosemary	0.002000	-0.2829 to 0.2869	No	ns	>0.9999	C-N
lemon vs. sage	0.03800	-0.2469 to 0.3229	No	ns	>0.9999	C-O
lemon vs. clove	-0.6300	-0.9149 to -0.3451	Yes	****	<0.0001	C-P
lemon vs. thyme	-1.199	-1.484 to -0.9141	Yes	****	<0.0001	C-Q
lemon vs. ginger	0.08800	-0.1969 to 0.3729	No	ns	0.9998	C-R
cumin vs. juniper	-0.2710	-0.5559 to 0.01392	No	ns	0.0842	D-E
cumin vs. lavender	-0.1000	-0.3849 to 0.1849	No	ns	0.9991	D-F
cumin vs. laurel	0.001000	-0.2839 to 0.2859	No	ns	>0.9999	D-G
cumin vs. tea tree	-0.6860	-0.9709 to -0.4011	Yes	****	<0.0001	D-H
cumin vs. peppermint	-0.3060	-0.5909 to -0.02108	Yes	*	0.0210	D-I
cumin vs. myrtle	0.08300	-0.2019 to 0.3679	No	ns	>0.9999	D-J
cumin vs. basil	0.1010	-0.1839 to 0.3859	No	ns	0.9990	D-K
cumin vs. oregano	-1.495	-1.780 to -1.210	Yes	****	<0.0001	D-L
cumin vs. black pepper	-0.01100	-0.2959 to 0.2739	No	ns	>0.9999	D-M
cumin vs. rosemary	0.05600	-0.2289 to 0.3409	No	ns	>0.9999	D-N
cumin vs. sage	0.09200	-0.1929 to 0.3769	No	ns	0.9997	D-O
cumin vs. clove	-0.5760	-0.8609 to -0.2911	Yes	****	<0.0001	D-P
cumin vs. thyme	-1.145	-1.430 to -0.8601	Yes	****	<0.0001	D-Q
cumin vs. ginger	0.1420	-0.1429 to 0.4269	No	ns	0.9568	D-R

juniper vs. lavender	0.1710	-0.1139 to 0.4559	No	ns	0.8140	E-F
juniper vs. laurel	0.2720	-0.01292 to 0.5569	No	ns	0.0812	E-G
juniper vs. tea tree	-0.4150	-0.6999 to -0.1301	Yes	****	<0.0001	E-H
juniper vs. peppermint	-0.03500	-0.3199 to 0.2499	No	ns	>0.9999	E-I
juniper vs. myrtle	0.3540	0.06908 to 0.6389	Yes	**	0.0021	E-J
juniper vs. basil	0.3720	0.08708 to 0.6569	Yes	***	0.0008	E-K
juniper vs. oregano	-1.224	-1.509 to -0.9391	Yes	****	<0.0001	E-L
juniper vs. black pepper	0.2600	-0.02492 to 0.5449	No	ns	0.1232	E-M
juniper vs. rosemary	0.3270	0.04208 to 0.6119	Yes	**	0.0081	E-N
juniper vs. sage	0.3630	0.07808 to 0.6479	Yes	**	0.0013	E-O
juniper vs. clove	-0.3050	-0.5899 to -0.02008	Yes	*	0.0219	E-P
juniper vs. thyme	-0.8740	-1.159 to -0.5891	Yes	****	<0.0001	E-Q
juniper vs. ginger	0.4130	0.1281 to 0.6979	Yes	****	<0.0001	E-R
lavender vs. laurel	0.1010	-0.1839 to 0.3859	No	ns	0.9990	F-G
lavender vs. tea tree	-0.5860	-0.8709 to -0.3011	Yes	****	<0.0001	F-H
lavender vs. peppermint	-0.2060	-0.4909 to 0.07892	No	ns	0.5059	F-I
lavender vs. myrtle	0.1830	-0.1019 to 0.4679	No	ns	0.7188	F-J
lavender vs. basil	0.2010	-0.08392 to 0.4859	No	ns	0.5532	F-K
lavender vs. oregano	-1.395	-1.680 to -1.110	Yes	****	<0.0001	F-L
lavender vs. black pepper	0.08900	-0.1959 to 0.3739	No	ns	0.9998	F-M
lavender vs. rosemary	0.1560	-0.1289 to 0.4409	No	ns	0.9042	F-N
lavender vs. sage	0.1920	-0.09292 to 0.4769	No	ns	0.6379	F-O
lavender vs. clove	-0.4760	-0.7609 to -0.1911	Yes	****	<0.0001	F-P
lavender vs. thyme	-1.045	-1.330 to -0.7601	Yes	****	<0.0001	F-Q
lavender vs. ginger	0.2420	-0.04292 to 0.5269	No	ns	0.2153	F-R
laurel vs. tea tree	-0.6870	-0.9719 to -0.4021	Yes	****	<0.0001	G-H
laurel vs. peppermint	-0.3070	-0.5919 to -0.02208	Yes	*	0.0201	G-I
laurel vs. myrtle	0.08200	-0.2029 to 0.3669	No	ns	>0.9999	G-J
laurel vs. basil	0.1000	-0.1849 to 0.3849	No	ns	0.9991	G-K
laurel vs. oregano	-1.496	-1.781 to -1.211	Yes	****	<0.0001	G-L
laurel vs. black pepper	-0.01200	-0.2969 to 0.2729	No	ns	>0.9999	G-M
laurel vs. rosemary	0.05500	-0.2299 to 0.3399	No	ns	>0.9999	G-N
laurel vs. sage	0.09100	-0.1939 to 0.3759	No	ns	0.9997	G-O
laurel vs. clove	-0.5770	-0.8619 to -0.2921	Yes	****	<0.0001	G-P
laurel vs. thyme	-1.146	-1.431 to -0.8611	Yes	****	<0.0001	G-Q
laurel vs. ginger	0.1410	-0.1439 to 0.4259	No	ns	0.9595	G-R
tea tree vs. peppermint	0.3800	0.09508 to 0.6649	Yes	***	0.0005	H-I
tea tree vs. myrtle	0.7690	0.4841 to 1.054	Yes	****	<0.0001	H-J
tea tree vs. basil	0.7870	0.5021 to 1.072	Yes	****	<0.0001	H-K
tea tree vs. oregano	-0.8090	-1.094 to -0.5241	Yes	****	<0.0001	H-L
tea tree vs. black pepper	0.6750	0.3901 to 0.9599	Yes	****	<0.0001	H-M
tea tree vs. rosemary	0.7420	0.4571 to 1.027	Yes	****	<0.0001	H-N
tea tree vs. sage	0.7780	0.4931 to 1.063	Yes	****	<0.0001	H-O
tea tree vs. clove	0.1100	-0.1749 to 0.3949	No	ns	0.9970	H-P
tea tree vs. thyme	-0.4590	-0.7439 to -0.1741	Yes	****	<0.0001	H-Q
tea tree vs. ginger	0.8280	0.5431 to 1.113	Yes	****	<0.0001	H-R
peppermint vs. myrtle	0.3890	0.1041 to 0.6739	Yes	***	0.0003	I-J
peppermint vs. basil	0.4070	0.1221 to 0.6919	Yes	***	0.0001	I-K
peppermint vs. oregano	-1.189	-1.474 to -0.9041	Yes	****	<0.0001	I-L
peppermint vs. black pepper	0.2950	0.01008 to 0.5799	Yes	*	0.0334	I-M
peppermint vs. rosemary	0.3620	0.07708 to 0.6469	Yes	**	0.0014	I-N
peppermint vs. sage	0.3980	0.1131 to 0.6829	Yes	***	0.0002	I-O
peppermint vs. clove	-0.2700	-0.5549 to 0.01492	No	ns	0.0873	I-P
peppermint vs. thyme	-0.8390	-1.124 to -0.5541	Yes	****	<0.0001	I-Q
peppermint vs. ginger	0.4480	0.1631 to 0.7329	Yes	****	<0.0001	I-R
myrtle vs. basil	0.01800	-0.2669 to 0.3029	No	ns	>0.9999	J-K
myrtle vs. oregano	-1.578	-1.863 to -1.293	Yes	****	<0.0001	J-L
myrtle vs. black pepper	-0.09400	-0.3789 to 0.1909	No	ns	0.9996	J-M
myrtle vs. rosemary	-0.02700	-0.3119 to 0.2579	No	ns	>0.9999	J-N
myrtle vs. sage	0.009000	-0.2759 to 0.2939	No	ns	>0.9999	J-O
myrtle vs. clove	-0.6590	-0.9439 to -0.3741	Yes	****	<0.0001	J-P
myrtle vs. thyme	-1.228	-1.513 to -0.9431	Yes	****	<0.0001	J-Q
myrtle vs. ginger	0.05900	-0.2259 to 0.3439	No	ns	>0.9999	J-R
basil vs. oregano	-1.596	-1.881 to -1.311	Yes	****	<0.0001	K-L
basil vs. black pepper	-0.1120	-0.3969 to 0.1729	No	ns	0.9964	K-M
basil vs. rosemary	-0.04500	-0.3299 to 0.2399	No	ns	>0.9999	K-N
basil vs. sage	-0.009000	-0.2939 to 0.2759	No	ns	>0.9999	K-O
basil vs. clove	-0.6770	-0.9619 to -0.3921	Yes	****	<0.0001	K-P
basil vs. thyme	-1.246	-1.531 to -0.9611	Yes	****	<0.0001	K-Q
basil vs. ginger	0.04100	-0.2439 to 0.3259	No	ns	>0.9999	K-R
oregano vs. black pepper	1.484	1.199 to 1.769	Yes	****	<0.0001	L-M
oregano vs. rosemary	1.551	1.266 to 1.836	Yes	****	<0.0001	L-N
oregano vs. sage	1.587	1.302 to 1.872	Yes	****	<0.0001	L-O
oregano vs. clove	0.9190	0.6341 to 1.204	Yes	****	<0.0001	L-P
oregano vs. thyme	0.3500	0.06508 to 0.6349	Yes	**	0.0026	L-Q
oregano vs. ginger	1.637	1.352 to 1.922	Yes	****	<0.0001	L-R
black pepper vs. rosemary	0.06700	-0.2179 to 0.3519	No	ns	>0.9999	M-N
black pepper vs. sage	0.1030	-0.1819 to 0.3879	No	ns	0.9987	M-O

black pepper vs. clove	-0.5650	-0.8499 to -0.2801	Yes	****	<0.0001	M-P
black pepper vs. thyme	-1.134	-1.419 to -0.8491	Yes	****	<0.0001	M-Q
black pepper vs. ginger	0.1530	-0.1319 to 0.4379	No	ns	0.9179	M-R
rosemary vs. sage	0.03600	-0.2489 to 0.3209	No	ns	>0.9999	N-O
rosemary vs. clove	-0.6320	-0.9169 to -0.3471	Yes	****	<0.0001	N-P
rosemary vs. thyme	-1.201	-1.486 to -0.9161	Yes	****	<0.0001	N-Q
rosemary vs. ginger	0.08600	-0.1989 to 0.3709	No	ns	0.9999	N-R
sage vs. clove	-0.6680	-0.9529 to -0.3831	Yes	****	<0.0001	O-P
sage vs. thyme	-1.237	-1.522 to -0.9521	Yes	****	<0.0001	O-Q
sage vs. ginger	0.05000	-0.2349 to 0.3349	No	ns	>0.9999	O-R
clove vs. thyme	-0.5690	-0.8539 to -0.2841	Yes	****	<0.0001	P-Q
clove vs. ginger	0.7180	0.4331 to 1.003	Yes	****	<0.0001	P-R
thyme vs. ginger	1.287	1.002 to 1.572	Yes	****	<0.0001	Q-R

Printout S2. The main effect of EO exposure on mean hd measurements in Gram-negative bacteria. Between-group analysis for the effect of EO exposure was performed by one-way ANOVA, followed by a post-hoc Tukey's multiple comparisons test.

ANOVA results | **Multiple comparisons**

Ordinary one-way ANOVA<br>Multiple comparisons						
Number of families	1					
Number of comparisons per family	153					
Alpha	0.05					
**Tukey's multiple comparisons test**	**Mean Diff.**	**95.00% CI of diff.**	**Below threshold?**	**Summary**	**Adjusted P Value**	
cinnamon vs. bergamot	0.5980	0.1605 to 1.035	Yes	***	0.0005	A-B
cinnamon vs. lemon	0.5980	0.1605 to 1.035	Yes	***	0.0005	A-C
cinnamon vs. cumin	0.5980	0.1605 to 1.035	Yes	***	0.0005	A-D
cinnamon vs. juniper	0.4650	0.02750 to 0.9025	Yes	*	0.0250	A-E
cinnamon vs. lavender	0.3890	-0.04850 to 0.8265	No	ns	0.1463	A-F
cinnamon vs. laurel	0.4600	0.02250 to 0.8975	Yes	*	0.0285	A-G
cinnamon vs. tea tree	-0.4060	-0.8435 to 0.03150	No	ns	0.1028	A-H
cinnamon vs. peppermint	0.4190	-0.01850 to 0.8565	No	ns	0.0771	A-I
cinnamon vs. myrtle	0.5350	0.09750 to 0.9725	Yes	**	0.0034	A-J
cinnamon vs. basil	0.5980	0.1605 to 1.035	Yes	***	0.0005	A-K
cinnamon vs. oregano	-1.081	-1.518 to -0.6435	Yes	****	<0.0001	A-L
cinnamon vs. black pepper	0.5980	0.1605 to 1.035	Yes	***	0.0005	A-M
cinnamon vs. rosemary	0.4350	-0.002498 to 0.8725	No	ns	0.0531	A-N
cinnamon vs. sage	0.5980	0.1605 to 1.035	Yes	***	0.0005	A-O
cinnamon vs. clove	-0.1310	-0.5685 to 0.3065	No	ns	0.9998	A-P
cinnamon vs. thyme	-0.4600	-0.8975 to -0.02250	Yes	*	0.0285	A-Q
cinnamon vs. ginger	0.5980	0.1605 to 1.035	Yes	***	0.0005	A-R
bergamot vs. lemon	0.000	-0.4375 to 0.4375	No	ns	>0.9999	B-C
bergamot vs. cumin	0.000	-0.4375 to 0.4375	No	ns	>0.9999	B-D
bergamot vs. juniper	-0.1330	-0.5705 to 0.3045	No	ns	0.9998	B-E
bergamot vs. lavender	-0.2090	-0.6465 to 0.2285	No	ns	0.9628	B-F
bergamot vs. laurel	-0.1380	-0.5755 to 0.2995	No	ns	0.9996	B-G
bergamot vs. tea tree	-1.004	-1.441 to -0.5665	Yes	****	<0.0001	B-H
bergamot vs. peppermint	-0.1790	-0.6165 to 0.2585	No	ns	0.9920	B-I
bergamot vs. myrtle	-0.06300	-0.5005 to 0.3745	No	ns	>0.9999	B-J
bergamot vs. basil	0.000	-0.4375 to 0.4375	No	ns	>0.9999	B-K
bergamot vs. oregano	-1.679	-2.116 to -1.242	Yes	****	<0.0001	B-L
bergamot vs. black pepper	0.000	-0.4375 to 0.4375	No	ns	>0.9999	B-M

bergamot vs. peppermint	-0.1790	-0.6165 to 0.2585	No	ns	0.9920	B-I
bergamot vs. myrtle	-0.06300	-0.5005 to 0.3745	No	ns	>0.9999	B-J
bergamot vs. basil	0.000	-0.4375 to 0.4375	No	ns	>0.9999	B-K
bergamot vs. oregano	-1.679	-2.116 to -1.242	Yes	****	<0.0001	B-L
bergamot vs. black pepper	0.000	-0.4375 to 0.4375	No	ns	>0.9999	B-M
bergamot vs. rosemary	-0.1630	-0.6005 to 0.2745	No	ns	0.9972	B-N
bergamot vs. sage	0.000	-0.4375 to 0.4375	No	ns	>0.9999	B-O
bergamot vs. clove	-0.7290	-1.166 to -0.2915	Yes	****	<0.0001	B-P
bergamot vs. thyme	-1.058	-1.495 to -0.6205	Yes	****	<0.0001	B-Q
bergamot vs. ginger	0.000	-0.4375 to 0.4375	No	ns	>0.9999	B-R
lemon vs. cumin	0.000	-0.4375 to 0.4375	No	ns	>0.9999	C-D
lemon vs. juniper	-0.1330	-0.5705 to 0.3045	No	ns	0.9998	C-E
lemon vs. lavender	-0.2090	-0.6465 to 0.2285	No	ns	0.9628	C-F
lemon vs. laurel	-0.1380	-0.5755 to 0.2995	No	ns	0.9996	C-G
lemon vs. tea tree	-1.004	-1.441 to -0.5665	Yes	****	<0.0001	C-H
lemon vs. peppermint	-0.1790	-0.6165 to 0.2585	No	ns	0.9920	C-I
lemon vs. myrtle	-0.06300	-0.5005 to 0.3745	No	ns	>0.9999	C-J
lemon vs. basil	0.000	-0.4375 to 0.4375	No	ns	>0.9999	C-K
lemon vs. oregano	-1.679	-2.116 to -1.242	Yes	****	<0.0001	C-L
lemon vs. black pepper	0.000	-0.4375 to 0.4375	No	ns	>0.9999	C-M
lemon vs. rosemary	-0.1630	-0.6005 to 0.2745	No	ns	0.9972	C-N
lemon vs. sage	0.000	-0.4375 to 0.4375	No	ns	>0.9999	C-O
lemon vs. clove	-0.7290	-1.166 to -0.2915	Yes	****	<0.0001	C-P
lemon vs. thyme	-1.058	-1.495 to -0.6205	Yes	****	<0.0001	C-Q
lemon vs. ginger	0.000	-0.4375 to 0.4375	No	ns	>0.9999	C-R
cumin vs. juniper	-0.1330	-0.5705 to 0.3045	No	ns	0.9998	D-E
cumin vs. lavender	-0.2090	-0.6465 to 0.2285	No	ns	0.9628	D-F
cumin vs. laurel	-0.1380	-0.5755 to 0.2995	No	ns	0.9996	D-G
cumin vs. tea tree	-1.004	-1.441 to -0.5665	Yes	****	<0.0001	D-H
cumin vs. peppermint	-0.1790	-0.6165 to 0.2585	No	ns	0.9920	D-I
cumin vs. myrtle	-0.06300	-0.5005 to 0.3745	No	ns	>0.9999	D-J
cumin vs. basil	0.000	-0.4375 to 0.4375	No	ns	>0.9999	D-K
cumin vs. oregano	-1.679	-2.116 to -1.242	Yes	****	<0.0001	D-L
cumin vs. black pepper	0.000	-0.4375 to 0.4375	No	ns	>0.9999	D-M
cumin vs. rosemary	-0.1630	-0.6005 to 0.2745	No	ns	0.9972	D-N
cumin vs. sage	0.000	-0.4375 to 0.4375	No	ns	>0.9999	D-O
cumin vs. clove	-0.7290	-1.166 to -0.2915	Yes	****	<0.0001	D-P
cumin vs. thyme	-1.058	-1.495 to -0.6205	Yes	****	<0.0001	D-Q
cumin vs. ginger	0.000	-0.4375 to 0.4375	No	ns	>0.9999	D-R
juniper vs. lavender	-0.07600	-0.5135 to 0.3615	No	ns	>0.9999	E-F
juniper vs. laurel	-0.005000	-0.4425 to 0.4325	No	ns	>0.9999	E-G
juniper vs. tea tree	-0.8710	-1.308 to -0.4335	Yes	****	<0.0001	E-H
juniper vs. peppermint	-0.04600	-0.4835 to 0.3915	No	ns	>0.9999	E-I
juniper vs. myrtle	0.07000	-0.3675 to 0.5075	No	ns	>0.9999	E-J
juniper vs. basil	0.1330	-0.3045 to 0.5705	No	ns	0.9998	E-K
juniper vs. oregano	-1.546	-1.983 to -1.109	Yes	****	<0.0001	E-L
juniper vs. black pepper	0.1330	-0.3045 to 0.5705	No	ns	0.9998	E-M
juniper vs. rosemary	-0.03000	-0.4675 to 0.4075	No	ns	>0.9999	E-N
juniper vs. sage	0.1330	-0.3045 to 0.5705	No	ns	0.9998	E-O
juniper vs. clove	-0.5960	-1.033 to -0.1585	Yes	***	0.0005	E-P
juniper vs. thyme	-0.9250	-1.362 to -0.4875	Yes	****	<0.0001	E-Q
juniper vs. ginger	0.1330	-0.3045 to 0.5705	No	ns	0.9998	E-R
lavender vs. laurel	0.07100	-0.3665 to 0.5085	No	ns	>0.9999	F-G
lavender vs. tea tree	-0.7950	-1.232 to -0.3575	Yes	****	<0.0001	F-H
lavender vs. peppermint	0.03000	-0.4075 to 0.4675	No	ns	>0.9999	F-I
lavender vs. myrtle	0.1460	-0.2915 to 0.5835	No	ns	0.9993	F-J
lavender vs. basil	0.2090	-0.2285 to 0.6465	No	ns	0.9628	F-K
lavender vs. oregano	-1.470	-1.907 to -1.033	Yes	****	<0.0001	F-L
lavender vs. black pepper	0.2090	-0.2285 to 0.6465	No	ns	0.9628	F-M
lavender vs. rosemary	0.04600	-0.3915 to 0.4835	No	ns	>0.9999	F-N
lavender vs. sage	0.2090	-0.2285 to 0.6465	No	ns	0.9628	F-O
lavender vs. clove	-0.5200	-0.9575 to -0.08250	Yes	**	0.0054	F-P

lavender vs. thyme	-0.8490	-1.286 to -0.4115	Yes	****	<0.0001	F-Q
lavender vs. ginger	0.2090	-0.2285 to 0.6465	No	ns	0.9628	F-R
laurel vs. tea tree	-0.8660	-1.303 to -0.4285	Yes	****	<0.0001	G-H
laurel vs. peppermint	-0.04100	-0.4785 to 0.3965	No	ns	>0.9999	G-I
laurel vs. myrtle	0.07500	-0.3625 to 0.5125	No	ns	>0.9999	G-J
laurel vs. basil	0.1380	-0.2995 to 0.5755	No	ns	0.9996	G-K
laurel vs. oregano	-1.541	-1.978 to -1.104	Yes	****	<0.0001	G-L
laurel vs. black pepper	0.1380	-0.2995 to 0.5755	No	ns	0.9996	G-M
laurel vs. rosemary	-0.02500	-0.4625 to 0.4125	No	ns	>0.9999	G-N
laurel vs. sage	0.1380	-0.2995 to 0.5755	No	ns	0.9996	G-O
laurel vs. clove	-0.5910	-1.028 to -0.1535	Yes	***	0.0006	G-P
laurel vs. thyme	-0.9200	-1.357 to -0.4825	Yes	****	<0.0001	G-Q
laurel vs. ginger	0.1380	-0.2995 to 0.5755	No	ns	0.9996	G-R
tea tree vs. peppermint	0.8250	0.3875 to 1.262	Yes	****	<0.0001	H-I
tea tree vs. myrtle	0.9410	0.5035 to 1.378	Yes	****	<0.0001	H-J
tea tree vs. basil	1.004	0.5665 to 1.441	Yes	****	<0.0001	H-K
tea tree vs. oregano	-0.6750	-1.112 to -0.2375	Yes	****	<0.0001	H-L
tea tree vs. black pepper	1.004	0.5665 to 1.441	Yes	****	<0.0001	H-M
tea tree vs. rosemary	0.8410	0.4035 to 1.278	Yes	****	<0.0001	H-N
tea tree vs. sage	1.004	0.5665 to 1.441	Yes	****	<0.0001	H-O
tea tree vs. clove	0.2750	-0.1625 to 0.7125	No	ns	0.7237	H-P
tea tree vs. thyme	-0.05400	-0.4915 to 0.3835	No	ns	>0.9999	H-Q
tea tree vs. ginger	1.004	0.5665 to 1.441	Yes	****	<0.0001	H-R
peppermint vs. myrtle	0.1160	-0.3215 to 0.5535	No	ns	>0.9999	I-J
peppermint vs. basil	0.1790	-0.2585 to 0.6165	No	ns	0.9920	I-K
peppermint vs. oregano	-1.500	-1.937 to -1.063	Yes	****	<0.0001	I-L
peppermint vs. black pepper	0.1790	-0.2585 to 0.6165	No	ns	0.9920	I-M
peppermint vs. rosemary	0.01600	-0.4215 to 0.4535	No	ns	>0.9999	I-N
peppermint vs. sage	0.1790	-0.2585 to 0.6165	No	ns	0.9920	I-O
peppermint vs. clove	-0.5500	-0.9875 to -0.1125	Yes	**	0.0021	I-P
peppermint vs. thyme	-0.8790	-1.316 to -0.4415	Yes	****	<0.0001	I-Q
peppermint vs. ginger	0.1790	-0.2585 to 0.6165	No	ns	0.9920	I-R
myrtle vs. basil	0.06300	-0.3745 to 0.5005	No	ns	>0.9999	J-K
myrtle vs. oregano	-1.616	-2.053 to -1.179	Yes	****	<0.0001	J-L
myrtle vs. black pepper	0.06300	-0.3745 to 0.5005	No	ns	>0.9999	J-M
myrtle vs. rosemary	-0.1000	-0.5375 to 0.3375	No	ns	>0.9999	J-N
myrtle vs. sage	0.06300	-0.3745 to 0.5005	No	ns	>0.9999	J-O
myrtle vs. clove	-0.6660	-1.103 to -0.2285	Yes	****	<0.0001	J-P
myrtle vs. thyme	-0.9950	-1.432 to -0.5575	Yes	****	<0.0001	J-Q
myrtle vs. ginger	0.06300	-0.3745 to 0.5005	No	ns	>0.9999	J-R
basil vs. oregano	-1.679	-2.116 to -1.242	Yes	****	<0.0001	K-L
basil vs. black pepper	0.000	-0.4375 to 0.4375	No	ns	>0.9999	K-M
basil vs. rosemary	-0.1630	-0.6005 to 0.2745	No	ns	0.9972	K-N
basil vs. sage	0.000	-0.4375 to 0.4375	No	ns	>0.9999	K-O
basil vs. clove	-0.7290	-1.166 to -0.2915	Yes	****	<0.0001	K-P
basil vs. thyme	-1.058	-1.495 to -0.6205	Yes	****	<0.0001	K-Q
basil vs. ginger	0.000	-0.4375 to 0.4375	No	ns	>0.9999	K-R
oregano vs. black pepper	1.679	1.242 to 2.116	Yes	****	<0.0001	L-M
oregano vs. rosemary	1.516	1.079 to 1.953	Yes	****	<0.0001	L-N
oregano vs. sage	1.679	1.242 to 2.116	Yes	****	<0.0001	L-O
oregano vs. clove	0.9500	0.5125 to 1.387	Yes	****	<0.0001	L-P
oregano vs. thyme	0.6210	0.1835 to 1.058	Yes	***	0.0002	L-Q
oregano vs. ginger	1.679	1.242 to 2.116	Yes	****	<0.0001	L-R
black pepper vs. rosemary	-0.1630	-0.6005 to 0.2745	No	ns	0.9972	M-N
black pepper vs. sage	0.000	-0.4375 to 0.4375	No	ns	>0.9999	M-O
black pepper vs. clove	-0.7290	-1.166 to -0.2915	Yes	****	<0.0001	M-P
black pepper vs. thyme	-1.058	-1.495 to -0.6205	Yes	****	<0.0001	M-Q
black pepper vs. ginger	0.000	-0.4375 to 0.4375	No	ns	>0.9999	M-R
rosemary vs. sage	0.1630	-0.2745 to 0.6005	No	ns	0.9972	N-O
rosemary vs. clove	-0.5660	-1.003 to -0.1285	Yes	**	0.0013	N-P
rosemary vs. thyme	-0.8950	-1.332 to -0.4575	Yes	****	<0.0001	N-Q
rosemary vs. ginger	0.1630	-0.2745 to 0.6005	No	ns	0.9972	N-R
sage vs. clove	-0.7290	-1.166 to -0.2915	Yes	****	<0.0001	O-P
sage vs. thyme	-1.058	-1.495 to -0.6205	Yes	****	<0.0001	O-Q
sage vs. ginger	0.000	-0.4375 to 0.4375	No	ns	>0.9999	O-R
clove vs. thyme	-0.3290	-0.7665 to 0.1085	No	ns	0.4059	P-Q
clove vs. ginger	0.7290	0.2915 to 1.166	Yes	****	<0.0001	P-R
thyme vs. ginger	1.058	0.6205 to 1.495	Yes	****	<0.0001	Q-R

Table S1. Mean values and standard deviations of inhibition halo diameters of 18 EOs against 13 Gram-positive bacteria on BHI agar plates

		cinnamon	bergamot	lemon	cumin	juniper	lavender	laurel	tea tree	peppermint	myrtle	basil	oregano	black pepper	rosemary	sage	clove	thyme	ginger
E. faecalis ATCC 29212	**24 h**	0	0	0	0	0	0	0	0	0	0	0	1.53 ± 0.06	0	0	0	0.7 ± 0.2	1.23 ± 0.4	0
	**48 h**	0	0	0	0	0	0	0	0	0	0	0	1.57 ± 0.21	0	0	0	0.7 ± 0.1	1.17 ± 0.15	0
	**7 d**	0	0	0	0	0	0	0	0	0	0	0	1.57 ± 0.12	0	0	0	0.7 ± 0.17	1.13 ± 0.12	0
L. monocytogenes ATCC 19111	**24 h**	0.57 ± 0.06	0	0	0.5 ± 0.2	0.5 ± 0.17	0	0	0	0	0	0	1.43 ± 0.4	0	0	0	1.13 ± 0.35	1.63 ± 0.12	0
	**48 h**	0.53 ± 0.06	0	0	0	0	0	0	0	0	0	0	1.47 ± 0.42	0	0	0	1.13 ± 0.32	1.30 ± 0.17	0
	**7 d**	0.5 ± 0.0	0	0	0	0	0	0	0	0	0	0	1.47 ± 0.04	0	0	0	1.0 ± 0.36	1.20 ± 0.20	0
S. aureus ATCC 6538	**24 h**	0.73 ± 0.12	0	0	0.67 ± 0.29	0.7 ± 0.26	0	0	1.67 ± 0.15	1.03 ± 0.25	0	0	1.7 ± 0.1	0	0	0	1.0 ± 0.10	1.37 ± 0.23	0
	**48 h**	0.6 ± 0.0	0	0	0	0	0	0	1.27 ± 0.45	0.83 ± 0.15	0	0	1.03 ± 0.06	0	0	0	0.97 ± 0.06	1.27 ± 0.15	0
	**7 d**	0.6 ± 0.0	0	0	0	0	0	0	1.13 ± 0.31	0.8 ± 0.17	0	0	0.93 ± 0.06	0	0	0	0.83 ± 0.06	1.23 ± 0.12	0
S. aureus LMG 16805	**24 h**	0.8 ± 0.17	0	0	0	0.5 ± 0.0	0	0	1.4 ± 0.17	0	0.5 ± 0.0	0.5 ± 0.1	2.27 ± 0.32	0	0	0	1.0 ± 0.0	2.07 ± 0.42	0
	**48 h**	0.8 ± 0.17	0	0	0	0.5 ± 0.0	0	0	1.4 ± 0.17	0	0	0	2.0 ± 0.46	0	0	0	1.0 ± 0.0	1.83 ± 0.55	0
	**7 d**	0.77 ± 0.15	0	0	0	0	0	0	1.2 ± 0.17	0	0	0	1.97 ± 0.45	0	0	0	0.9 ± 0.17	1.7 ± 0.5	0

S. aureus SIC-11	**24 h**	0	0	0.53 ± 0.06	0	0	0.63 ± 0.06	0	0	0.6 ± 0.1	0	0	1.47 ± 0.06	0.57 ± 0.06	0	0	0.5 ± 0.0	0.97 ± 0.06	0
	**48 h**	0	0	0.53 ± 0.06	0	0	0.63 ± 0.06	0	0	0	0	0	1.47 ± 0.06	0.57 ± 0.06	0	0	0.5 ± 0.0	0.97 ± 0.06	0
	**7 d**	0	0	0.53 ± 0.06	0	0	0.63 ± 0.06	0	0	0	0	0	1.47 ± 0.06	0.57 ± 0.06	0	0	0.5 ± 0.0	0.97 ± 0.06	0
S. dysgalactiae SIC-10	**24 h**	0.57 ± 0.06	0	0	0	0.5 ± 0.0	0.73 ± 0.06	0	1.37 ± 0.12	0.83 ± 0.06	0	0	1.83 ± 0.47	0	0.5 ± 0.0	0	0.63 ± 0.12	1.20 ± 0.1	0
	**48 h**	0.57 ± 0.06	0	0	0	0.5 ± 0.0	0.73 ± 0.06	0	1.37 ± 0.12	0.8 ± 0	0	0	1.83 ± 0.47	0	0	0	0.63 ± 0.12	1.20 ± 0.1	0
	**7 d**	0.57 ± 0.06	0	0	0	0.5 ± 0.0	0.73 ± 0.06	0	1.37 ± 0.12	0.8 ± 0	0	0	1.57 ± 0.4	0	0	0	0.63 ± 0.12	1.20 ± 0.1	0
S. chromogenes SAR-15	**24 h**	0.6 ± 0.0	0	0	0	0.5 ± 0.0	0.73 ± 0.06	0	1.33 ± 0.06	1.37 ± 0.38	0	0	1.9 ± 0.2	0	0	0	0.87 ± 0.15	1.1 ± 0.1	0
	**48 h**	0.6 ± 0.0	0	0	0	0.5 ± 0.0	0.73 ± 0.06	0	1.1 ± 0.26	1.3 ± 0.26	0	0	1.6 ± 0.26	0	0	0	0.73 ± 0.06	1.07 ± 0.06	0
	**7 d**	0.6 ± 0.0	0	0	0	0.5 ± 0.06	0	0	0.97 ± 0.15	1.1 ± 0.26	0	0	1.53 ± 0.29	0	0	0	0.73 ± 0.06	1.07 ± 0.06	0
S. epidermidis SIC-14	**24 h**	0.87 ± 0.12	0	0	0.53 ± 0.06	0.5 ± 0.0	1.37 ± 0.06	1.23 ± 0.4	1.33 ± 0.15	1.5 ± 0.15	0	0	2.47 ± 0.21	0	0	0	0.9 ± 0.0	2.0 ± 0.0	0
	**48 h**	0.87 ± 0.12	0	0	0.53 ± 0.06	0.5 ± 0.0	1.37 ± 0.06	1.23 ± 0.4	1.3 ± 0.26	1.35 ± 0.26	0	0	2.17 ± 0.21	0	0	0	0.9 ± 0.0	2.0 ± 0.0	0
	**7 d**	0.87 ± 0.12	0	0	0.53 ± 0.06	0.5 ± 0.0	0	0.9 ± 0.17	1.23 ± 0.25	1.3 ± 0.26	0	0	2.07 ± 0.12	0	0	0	0.9 ± 0.0	2.0 ± 0.0	0
S. epidermidis SAR-16	**24 h**	0.5 ± 0.0	0	0	0	0	0.9 ± 0.17	0	0	0	0	0	1.73 ± 0.25	0.7 ± 0.1	0	0	0.5 ± 0.0	1.17 ± 0.06	0
	**48 h**	0.5 ± 0.0	0	0	0	0	0	0	0	0	0	0	1.73 ± 0.25	0.7 ± 0.1	0	0	0.5 ± 0.0	1.17 ± 0.06	0
	**7 d**	0.5 ± 0.0	0	0	0	0	0	0	0	0	0	0	1.73 ± 0.25	0.6 ± 0.1	0	0	0.5 ± 0.0	1.17 ± 0.06	0

S. uberis LMG 14750	**24 h**	0.57 ± 0.06	0	0	0	0.5 ± 0.0	0	0	1.63 ± 0.12	0	0	0	2.2 ± 0.7	0	0	0	0.87 ± 0.15	1.17 ± 0.12	0
	**48 h**	0.57 ± 0.06	0	0	0	0	0	0	1.57 ± 0.12	0	0	0	2.1 ± 0.61	0	0	0	0.87 ± 0.15	1.17 ± 0.12	0
	**7 d**	0.57 ± 0.06	0	0	0	0	0	0	1.57 ± 0.12	0	0	0	2.1 ± 0.61	0	0	0	0.87 ± 0.15	1.17 ± 0.12	0
S. agalactiae LMG 14838	**24 h**	0.7 ± 0.0	0	0	0	0	0	0	1.93 ± 0.47	0	0	0	1.97 ± 0.64	0	0	0	0.9 ± 0.29	1.57 ± 0.61	0
	**48 h**	0.7 ± 0.0	0	0	0	0	0	0	1.9 ± 0.44	0	0	0	1.83 ± 0.58	0	0	0	0.9 ± 0.29	1.57 ± 0.61	0
	**7 d**	0.63 ± 0.06	0	0	0	0	0	0	1.37 ± 0.12	0	0	0	1.83 ± 0.58	0	0	0	0.9 ± 0.29	1.57 ± 0.61	0
S. agalactiae SIC-12	**24 h**	0.77 ± 0.06	0	0.7 ± 0.0	0	1.27 ± 0.23	0.5 ± 0.0	0	0	1.0 ± 0.0	0	0	2.4 ± 0.36	0.77 ± 0.06	0	1.3 ± 0.44	0.87 ± 0.12	2.67 ± 0.29	0
	**48 h**	0.77 ± 0.06	0	0.7 ± 0.0	0	1.27 ± 0.23	0.5 ± 0.0	0	0	1.0 ± 0.0	0	0	2.4 ± 0.36	0.77 ± 0.06	0	1.3 ± 0.44	0.87 ± 0.12	2.67 ± 0.29	0
	**7 d**	0.63 ± 0.06	0	0.6 ± 0.0	0	1.23 ± 0.21	0.5 ± 0.06	0	0	1.0 ± 0.0	0	0	2.4 ± 0.36	0.7 ± 0.1	0	1.3 ± 0.44	0.87 ± 0.12	2.67 ± 0.29	0
S. intermedius SIC-8	**24 h**	0.73 ± 0.25	0	0	0	0	0	0	0.8 ± 0	0	0	0	2.0 ± 0.26	0	0	0	0.93 ± 0.06	1.4 ± 0.17	0
	**48 h**	0.7 ± 0.2	0	0	0	0	0	0	0.7 ± 0.1	0	0	0	1.97 ± 0.25	0	0	0	0.9 ± 0.1	1.27 ± 0.06	0
	**7 d**	0.7 ± 0.2	0	0	0	0	0	0	0.67 ± 0.06	0	0	0	1.9 ± 0.26	0	0	0	0.9 ± 0.1	1.27 ± 0.06	0

The mean values and standard deviations of the diameters of inhibition halos (hds) in centimetres are shown. The colours used to represent the EO's efficiency are red for very high antimicrobial activity (hd ≥2.0 cm), magenta for high antimicrobial activity (1.5≤ hd <2.0 cm), green for moderate antimicrobial activity (1.0≤ hd <1.5 cm), yellow for low antimicrobial activiy (0.8 ≤ hd <1.0 cm), and no colour for null or very low antimicrobial activity (hd < 0.8 cm).

Table S2. Mean values and standard deviations of inhibition halo diameters of 18 EOs against 13 Gram-positive bacteria on MH agar plates

		cinnamon	bergamot	lemon	cumin	juniper	lavender	laurel	tea tree	peppermint	myrtle	basil	oregano	black pepper	rosemary	sage	clove	thyme	ginger
E. faecalis ATCC 29212	**24 h**	0.53 ± 0.06	0	0	0	1.15 ± 0.21	0	0	0	0	0	0	0.67 ± 0.06	0	0	0	0	0.7 ± 0	0
	**48 h**	0	0	0	0	1.1 ± 0.14	0	0	0	0	0	0	0.67 ± 0.06	0	0	0	0	0.7 ± 0	0
	**7 d**	0	0	0	0	1.07 ± 0.23	0	0	0	0	0	0	0.67 ± 0.06	0	0	0	0	0.5 ± 0	0
L. monocytogenes ATCC 19111	**24 h**	0	0	0	0	0.7 ± 0.14	0	0	0	0.63 ± 0.12	0	0	0.87 ± 0.12	0	0	0	0	0.63 ± 0.12	0
	**48 h**	0	0	0	0	0.7 ± 0.14	0	0	0	0.53 ± 0.12	0	0	0.87 ± 0.12	0	0	0	0	0.63 ± 0.12	0
	**7 d**	0	0	0	0	0.6 ± 0.0	0	0	0	0	0	0	0.8 ± 0.1	0	0	0	0	0.63 ± 0.12	0
S. aureus ATCC 6538	**24 h**	0.9 ± 0.1	0	0.5 ± 0.1	0.5 ± 0.1	0.67 ± 0.06	0.63 ± 0.06	0	0.83 ± 0.25	0.87 ± 0.15	0	0	1.87 ± 0.15	0	0.57 ± 0.06	0	1.3 ± 0.3	1.1 ± 0.1	0
	**48 h**	0.9 ± 0.1	0	0.5 ± 0.1	0.5 ± 0.1	0	0	0	0.83 ± 0.06	0.8 ± 0.17	0	0	1.35 ± 0.21	0	0	0	1.23 ± 0.12	0.93 ± 0.12	0
	**7 d**	0.7 ± 0.06	0	0	0	0	0	0	0.73 ± 0.06	0.73 ± 0.21	0	0	1.1 ± 0.1	0	0	0	1.17 ± 0.06	0.87 ± 0.06	0
S. aureus LMG 16805	**24 h**	0.53 ± 0.12	0	0	0	0.5 ± 0.0	0	0.63 ± 0.21	1.1 ± 0.17	0.53 ± 0.12	0	0	1.3 ± 0.26	0	0.67 ± 0.21	0	0.6 ± 0	1.2 ± 0.1	0
	**48 h**	0.53 ± 0.12	0	0	0	0	0	0.53 ± 0.12	1.1 ± 0.17	0	0	0	1.3 ± 0.26	0	0.67 ± 0.21	0	0.53 ± 0.06	1.17 ± 0.12	0
	**7 d**	0	0	0	0	0	0	0.5 ± 0.2	1.1 ± 0.17	0	0	0	1.3 ± 0.26	0	0	0	0.5 ± 0.0	1.13 ± 0.15	0

S. aureus SIC-11	**24 h**	0.6 ± 0.1	0	0.57 ± 0.12	0	1.13 ± 0.12	0.8 ± 0.1	0	0.6 ± 0.1	1.07 ± 0.12	0.5 ± 0.0	0	1.6 ± 0.1	0.93 ± 0.06	0	0	0.5 ± 0.0	0.87 ± 0.12	0
	**48 h**	0.6 ± 0.1	0	0.57 ± 0.12	0	1.13 ± 0.12	0.8 ± 0.1	0	0.6 ± 0.1	1.07 ± 0.12	0	0	1.6 ± 0.1	0.93 ± 0.06	0	0	0.5 ± 0.0	0.87 ± 0.12	0
	**7 d**	0.6 ± 0.1	0	0.57 ± 0.12	0	1.13 ± 0.12	0.77 ± 0.15	0	0.6 ± 0.1	1.07 ± 0.12	0	0	1.6 ± 0.1	0.93 ± 0.06	0	0	0.5 ± 0.0	0.87 ± 0.12	0
S. dysgalactiae SIC-10	**24 h**	0.73 ± 0.12	0	0	0	0	0	0	0.53 ± 0.06	0.5 ± 0.0	0	0	1.53 ± 0.06	0	0.5 ± 0.1	0	0.87 ± 0.06	1.23 ± 0.12	0
	**48 h**	0.73 ± 0.12	0	0	0	0	0	0	0.53 ± 0.06	0.5 ± 0.0	0	0	1.53 ± 0.06	0	0.5 ± 0.1	0	0.8 ± 0.1	1.23 ± 0.12	0
	**7 d**	0.7 ± 0.1	0	0	0	0	0	0	0.53 ± 0.06	0.5 ± 0.0	0	0	1.3 ± 0.26	0	0	0	0.77 ± 0.12	1.17 ± 0.15	0
S. chromogenes SAR-15	**24 h**	0.6 ± 0.1	0	0	0.5 ± 0.0	0.5 ± 0.0	0	0.6 ± 0.1	1.67 ± 0.15	1.0 ± 0.0	0	0	1.07 ± 0.12	0	0	0	0.7 ± 0.1	0.67 ± 0.15	0
	**48 h**	0.6 ± 0.1	0	0	0	0.5 ± 0.0	0	0.57 ± 0.12	1.67 ± 0.15	1.0 ± 0.0	0	0	1.07 ± 0.12	0	0	0	0.7 ± 0.1	0.67 ± 0.15	0
	**7 d**	0.6 ± 0.1	0	0	0	0	0	0.57 ± 0.12	1.5 ± 0.3	1.0 ± 0.0	0	0	1.07 ± 0.12	0	0	0	0.65 ± 0.07	0.53 ± 0.06	0
S. epidermidis SIC-14	**24 h**	0.7 ± 0.0	0	0	0.5 ± 0.2	0	0	0.57 ± 0.23	1.33 ± 0.12	0.73 ± 0.06	0	0	2.33 ± 0.21	0	0	0	0.67 ± 0.06	1.83 ± 0.25	0
	**48 h**	0.7 ± 0.0	0	0	0.5 ± 0.2	0	0	0.57 ± 0.23	1.17 ± 0.15	0.73 ± 0.06	0	0	2.1 ± 0.36	0	0	0	0.67 ± 0.06	1.83 ± 0.25	0
	**7 d**	0.7 ± 0.0	0	0	0.5 ± 0.2	0	0	0	1.17 ± 0.15	0.73 ± 0.06	0	0	2.1 ± 0.36	0	0	0	0.67 ± 0.06	1.8 ± 0.1	0
S. epidermidis SAR-16	**24 h**	0	0	0	0	0	0	0	0.5 ± 0.0	0	0	0	1.47 ± 0.29	0	0	0	0.63 ± 0.06	1.27 ± 0.25	0
	**48 h**	0	0	0	0	0	0	0	0.5 ± 0.0	0	0	0	1.47 ± 0.29	0	0	0	0.63 ± 0.06	1.27 ± 0.25	0

	**7 d**	0	0	0	0	0	0	0	0.5 ± 0.0	0	0	0	1.27 ± 0.38	0	0	0	0.63 ± 0.06	1.27 ± 0.25	0
S. uberis LMG 14750	**24 h**	0.57 ± 0.06	0	0	0	0	0	0	1.43 ± 0.06	0	0.53 ± 0.15	0	1.4 ± 0.1	0	0	0	0.63 ± 0.06	1.07 ± 0.12	0
	**48 h**	0.57 ± 0.06	0	0	0	0	0	0	1.43 ± 0.06	0	0.53 ± 0.15	0	1.4 ± 0.1	0	0	0	0.63 ± 0.06	0.93 ± 0.32	0
	**7 d**	0.57 ± 0.06	0	0	0	0	0	0	1.43 ± 0.06	0	0.5 ± 0.1	0	1.4 ± 0.1	0	0	0	0.63 ± 0.06	0.93 ± 0.15	0
S. agalactiae LMG 14838	**24 h**	0.7 ± 0.1	0	0	0	0	0	0.63 ± 0.23	1.27 ± 0.21	0	0	0.57 ± 0.06	1.47 ± 0.21	0	0	0	0.63 ± 0.21	1.9 ± 0.4	0
	**48 h**	0.7 ± 0.1	0	0	0	0	0	0	1.27 ± 0.21	0	0	0.57 ± 0.06	1.47 ± 0.21	0	0	0	0.63 ± 0.21	1.53 ± 0.23	0
	**7 d**	0.63 ± 0.12	0	0	0	0	0	0	1.17 ± 0.15	0	0	0.57 ± 0.06	1.37 ± 0.12	0	0	0	0.63 ± 0.21	1.5 ± 0.23	0
S. agalactiae SIC-12	**24 h**	0	0	0	0.5 ± 0.2	1.13 ± 0.15	0	0	0	0	0	0	0.9 ± 0.0	1.0 ± 0.0	0	0	0.7 ± 0.0	0.73 ± 0.06	0
	**48 h**	0	0	0	0.5 ± 0.2	1.13 ± 0.15	0	0	0	0	0	0	0.9 ± 0.0	1.0 ± 0.0	0	0	0	0.73 ± 0.06	0
	**7 d**	0	0	0	0.5 ± 0.2	1.13 ± 0.15	0	0	0	0	0	0	0.9 ± 0.0	1.0 ± 0.0	0	0	0	0.73 ± 0.06	0
S. intermedius SIC-8	**24 h**	0.5 ± 0.3	0	0	0	0	0	0	0.8 ± 0.1	0	0	0	1.17 ± 0.12	0	0	0	0.63 ± 0.15	0.7 ± 0.06	0
	**48 h**	0.5 ± 0.26	0	0	0	0	0	0	0.8 ± 0.1	0	0	0	1.03 ± 0.06	0	0	0	0.63 ± 0.06	0.7 ± 0.06	0
	**7 d**	0.5 ± 0.26	0	0	0	0	0	0	0.8 ± 0.1	0	0	0	1.0 ± 0.14	0	0	0	0.6 ± 0.1	0.63 ± 0.06	0

The mean values and standard deviations of the diameters of inhibition halos (hds) in centimetres are shown. The colours used to represent the EO's efficiency are red for very high antimicrobial activity (hd ≥2.0 cm), magenta for high antimicrobial activity (1.5≤ hd <2.0 cm), green for moderate antimicrobial activity (1.0≤ hd <1.5 cm), yellow for low antimicrobial activiy (0.8 ≤ hd <1.0 cm), and no colour for null or very low antimicrobial activity (hd < 0.8 cm).

Table S3. Mean values and standard deviations of inhibition halo diameters of 18 EOs against four Gram-negative bacteria on BHI agar plates

			cinnamon	bergamot	lemon	cumin	juniper	lavender	laurel	tea tree	peppermint	myrtle	basil	oregano	black pepper	rosemary	sage	clove	thyme	ginger
S. enterica	ATCC 14028	**24 h**	0.53 ± 0.06	0	0	0	0	0	0	0.87 ± 0.06	0	0	0	1.6 ± 0.17	0	0	0	0.73 ± 0.06	0.93 ± 0.12	0
		**48 h**	0.53 ± 0.06	0	0	0	0	0	0	0.83 ± 0.06	0	0	0	1.6 ± 0.17	0	0	0	0.63 ± 0.06	0.93 ± 0.12	0
		**7 d**	0	0	0	0	0	0	0	0.83 ± 0.06	0	0	0	1.43 ± 0.06	0	0	0	0.63 ± 0.06	0.87 ± 0.06	0
S. enterica	NCTC 13347	**24 h**	0.5 ± 0	0	0	0	0.53 ± 0.06	0	0	0.5 ± 0.14	0	0	0	1.5 ± 0.1	0	0	0	0.7 ± 0.17	0.7 ± 0	0
		**48 h**	0	0	0	0	0	0	0	0.5 ± 0.14	0	0	0	1.4 ± 0.1	0	0	0	0.7 ± 0.17	0.67 ± 0.06	0
		**7 d**	0	0	0	0	0	0	0	0.5 ± 0.1	0	0	0	1.33 ± 0.15	0	0	0	0.67 ± 0.25	0.67 ± 0.06	0
E. coli	ATCC 25922	**24 h**	0.87 ± 0.06	0	0	0	0.53 ± 0.12	1.1 ± 0.32	0.53 ± 0.06	1.73 ± 0.32	1.43 ± 0.32	0	0	1,7 ± 0.25	0	0.7 ± 0.1	0	1.0 ± 0	1.53 ± 0.15	0
		**48 h**	0.87 ± 0.06	0	0	0	0.53 ± 0.12	1.1 ± 0.32	0.5 ± 0	1.73 ± 0.32	1.43 ± 0.32	0	0	1.67 ± 0.29	0	0.7 ± 0.1	0	1.0 ± 0	1.5 ± 0	0
		**7 d**	0.7 ± 0	0	0	0	0.53 ± 0.12	0.9 ± 0.17	0.5 ± 0	1.73 ± 0.32	1.43 ± 0.32	0	0	1.33 ± 0.58	0	0.7 ± 0.1	0	0.9 ± 0.1	1.5 ± 0	0
E. coli	SIC-9	**24 h**	0.5 ± 0.1	0	0	0	0	0	0	0.7 ± 0.17	0	0	0	2.2 ± 0.17	0	0	0	0.67 ± 0.31	1.43 ± 0.21	0
		**48 h**	0	0	0	0	0	0	0	0.7 ± 0.17	0	0	0	2.17 ± 0.15	0	0	0	0.67 ± 0.31	1.43 ± 0.21	0
		**7 d**	0	0	0	0	0	0	0	0.7 ± 0.17	0	0	0	2.07 ± 0.12	0	0	0	0.67 ± 0.31	1.43 ± 0.21	0

The mean values and standard deviations of the diameters of inhibition halos (hds) in centimetres are shown. The colours used to represent the EO's efficiency are red for very high antimicrobial activity (hd ≥2.0 cm), magenta for high antimicrobial activity (1.5≤ hd <2.0 cm), green for moderate antimicrobial activity (1.0≤ hd <1.5 cm), yellow for low antimicrobial activiy (0.8 ≤ hd <1.0 cm), and no colour for null or very low antimicrobial activity (hd < 0.8 cm).

Table S4. Mean values and standard deviations of inhibition halo diameters of 18 EOs against four Gram-negative bacteria on MH agar plates

			cinnamon	bergamot	lemon	cumin	juniper	lavender	laurel	tea tree	peppermint	myrtle	basil	oregano	black pepper	rosemary	sage	clove	thyme	ginger
S. enterica	ATCC 14028	**24 h**	0	0	0	0	0	0	0	0.8 ± 0.0	0	0	0	1.73 ± 0.21	0	0	0	0.63 ± 0.06	0.87 ± 0.06	0
		**48 h**	0	0	0	0	0	0	0	0.8 ± 0.0	0	0	0	1.5 ± 0.2	0	0	0	0.63 ± 0.06	0.73 ± 0.06	0
		**7 d**	0	0	0	0	0	0	0	0.8 ± 0.0	0	0	0	1.33 ± 0.15	0	0	0	0.6 ± 0.1	0.73 ± 0.06	0
S. enterica	NCTC 13347	**24 h**	0.93 ± 0.06	0	0	0	0	0	0	1.07 ± 0.06	0	0	0	1.3 ± 0.1	0	0	0	0.7 ± 0.0	0,87 ± 0.12	0
		**48 h**	0.8 ± 0.17	0	0	0	0	0	0	1.07 ± 0.06	0	0	0	1.3 ± 0.1	0	0	0	0.7 ± 0.0	0.87 ± 0.12	0
		**7 d**	0.8 ± 0.17	0	0	0	0	0	0	1.03 ± 0.12	0	0	0	1.3 ± 0.1	0	0	0	0.7 ± 0.0	0.87 ± 0.12	0
E. coli	ATCC 25922	**24 h**	0.6 ± 0.0	0	0	0	0	0.57 ± 0.31	0.57 ± 0.06	1.33 ± 0.06	0	0.5 ± 0.0	0	1.9 ± 0.36	0	0.6 ± 0.0	0	0.67 ± 0.06	1.13 ± 0.21	0
		**48 h**	0.6 ± 0.0	0	0	0	0	0	0.57 ± 0.06	1.3 ± 0.1	0	0.5 ± 0.0	0	1.9 ± 0.36	0	0	0	0.67 ± 0.06	1.13 ± 0.15	0
		**7 d**	0.5 ± 0.0	0	0	0	0	0	0.57 ± 0.06	1.27 ± 0.06	0	0.5 ± 0.0	0	1.9 ± 0.36	0	0	0	0.53 ± 0.06	1.07 ± 0.15	0

E. coli SIC-9	**24 h**	0.85 ± 0.07	0	0	0	0	0	0	1.03 ± 0.06	0	0	0	1.5 ± 0.12	0	0	0	0.73 ± 0.1	1.0 ± 0	0
	**48 h**	0.85 ± 0.07	0	0	0	0	0	0	1.03 ± 0.06	0	0	0	1.47 ± 0.29	0	0	0	0.7 ± 0.17	0.97 ± 0.06	0
	**7 d**	0.73 ± 0.06	0	0	0	0	0	0	1.03 ± 0.06	0	0	0	1.47 ± 0.29	0	0	0	0.7 ± 0.17	0.97 ± 0.06	0

The mean values and standard deviations of the diameters of inhibition halos (hds) in centimetres are shown. The colours used to represent the EO's efficiency are red for very high antimicrobial activity (hd ≥2.0 cm), magenta for high antimicrobial activity (1.5≤ hd <2.0 cm), green for moderate antimicrobial activity (1.0≤ hd <1.5 cm), yellow for low antimicrobial activiy (0.8 ≤ hd <1.0 cm), and no colour for null or very low antimicrobial activity (hd < 0.8 cm).
